# Artificial turf and crumb rubber infill: An international policy review concerning the current state of regulations

**DOI:** 10.1016/j.envc.2022.100620

**Published:** 2022-09-16

**Authors:** Philip Zuccaro, David C. Thompson, Jacob de Boer, Andrew Watterson, Qiong Wang, Song Tang, Xiaoming Shi, Maria Llompart, Nuno Ratola, Vasilis Vasiliou

**Affiliations:** a Yale University, New Haven, CT, USA; b Department of Environmental Health Sciences, Yale School of Public Health, New Haven, CT, USA; c Department of Environment and Health, Vrije Universiteit Amsterdam, Amsterdam, the Netherlands; d Faculty of Health Sciences and Sport, University of Stirling, Stirling, Scotland; e China CDC Key Laboratory of Environment and Population Health, National Institute of Environmental Health, Chinese Center for Disease Control and Prevention, Beijing, China; f Center for Global Health, School of Public Health, Nanjing Medical University, Nanjing, Jiangsu, China; g CRETUS, Department of Analytical Chemistry, Nutrition, and Food Sciences, Universidade de Santiago de Compostela, Santiago de Compostela, Spain; h LEPABE- Laboratory for Process Engineering, Environment, Biotechnology, and Energy, Faculty of Engineering, University of Porto, Porto, Portugal; i ALiCE - Associate Laboratory in Chemical Engineering, Faculty of Engineering, University of Porto, Porto, Portugal

**Keywords:** Artificial turf, Crumb rubber, Policy, Infill

## Abstract

**Background:**

Although artificial turf fields are utilized widely around the world, sufficient research has not yet been conducted to assess the potential human and environmental health risks posed by the chemicals contained in the fields’ fibers, backing, and often-used crumb rubber infill. Consequently, there is wide variation in governmental policies.

**Objective:**

Review the notable policies concerning artificial turf and crumb rubber infill in the European Union, United Kingdom, United States of America, Canada, China, Qatar, and the Global Stockholm Convention of the United Nations.

**Methods:**

Information was collected that included published papers, technical and policy reports, and grey literature. These were then analyzed by a collaborative group familiar with the environmental policies in their respective countries to extract the pertinent legislative or regulatory information. The group members were primarily identified through their involvement in publications pertinent to artificial turf and crumb rubber infill health research and included environmental health professors, active researchers, and governmental agency officials. Most information on direct policies was taken directly from reports provided to the public by various governmental agencies responsible for their countries’ regulations, often available within the respective agency’s online archives.

**Results:**

There are significant differences in the regulatory approaches adopted by the investigated countries with regards to artificial turf and its crumb rubber infill. Some regions, such as the European Union, have taken substantial steps to limit the fields’ chemical components to which the public and environment are exposed. Other regions and countries have done far less to address the issue. Most policies relate directly to (i) the fields themselves, (ii) the microplastic components of crumb rubber infill, or (iii) the concentrations of harmful polycyclic aromatic hydrocarbons (PAHs), perfluoroalkyl and polyfluoroalkyl substances (PFAS), and heavy metals.

**Conclusion:**

While nearly every country acknowledges the potential health risks posed by heavy metals, microplastics, PAHs, and PFAS chemicals, very few have actually implemented artificial turf and crumb rubber infill regulations and/or established adequate surveillance measures to protect those regularly exposed to the fields.

## Introduction

**Fig. 1.**
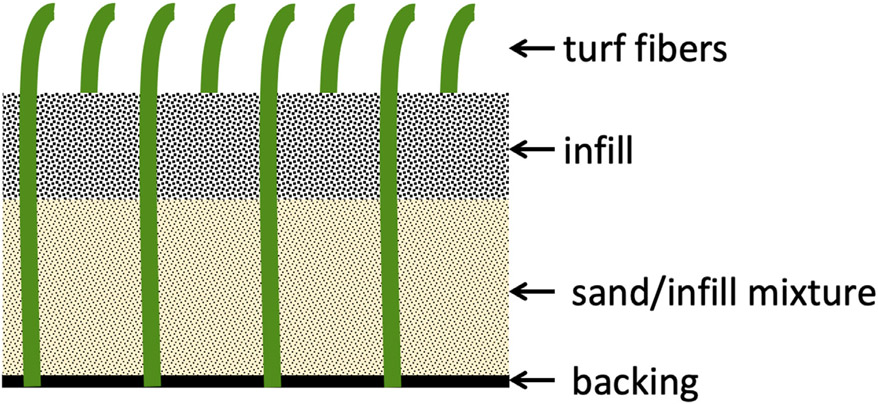
Artificial turf field components. Turf fibers (the blades of “grass”) are woven into backing material. Upon installation, an infill material is raked into the fibers to provide support. Oftentimes, a base of sand mixed with infill material is first applied, followed by a layer of infill material. Crumb rubber is a common infill material.

The European Chemicals Agency (ECHA) reported that in 2012 the European Union (EU) was home to at least 13,000 full-sized artificial turf fields and at least 45,000 smaller fields (Committee for Risk Assessment, Committee for Socio-Economic Analysis, 2019). It is further estimated that, as of 2020, the United States of America (USA) contained ≈13,000 artificial turf fields, with the total number for both regions expected to rise every year (Synthetic Turf Council, 2021). Although artificial turf fields have many components, the three pertinent parts to be considered for an understanding of existing policy and research are the backing, infill, and turf fibers (also referred to as yarn). Many different material options exist for use as artificial turf infill including thermoplastics, coconut fibers, cork, sand, and elastomer polymers. However, the most widespread type of turf infill by far is the recycled and shred-ded end-of-life tires referred to as crumb rubber (Committee for Risk Assessment, Committee for Socio-Economic Analysis, 2019). The turf fibers are the synthetic blades of “grass” and the backing is the underlying layer on which the infill sits and the turf fibers are connected (Fig. 1) (Purchase Green Artificial Turf, 2021).

There is currently much debate over the human and environmental health risks posed by the use of these artificial fields due to evidence of harmful chemicals contained in the artificial turf fibers and in the crumb rubber infill. A recent study from Yale University concluded that there are at least 306 different chemical agents in the crumb rubber infill, with as many as 197 exhibiting carcinogenic characteristics (Perkins et al., 2019). It was also revealed in the same study that as many as 207 of these crumb rubber chemicals were not officially catalogued by the United States Environmental Protection Agency (US EPA), thereby indicating incomplete documentation of the potential health effects (Perkins et al., 2019). Despite the global utilization of these artificial turf fields, insufficient research has been conducted to reach concluions regarding the human health impact caused by regular exposure to the chemicals contained therein or emitted therefrom. In the limited research that has been conducted, the health risk assessment studies have mostly been small in scale, indirect in risk determination, and lacking data on long-term human health impact. Of those studies conducted, many provide conflicting conclusions. A large number will point to findings that the artificial turf fields pose a potential risk to human health that should not be ignored, while many others claim that the artificial turf fields present little to no human health risks. For example, a risk assessment by the Connecticut Department of Public Health in 2010 concluded that (i) the chemicals found in air emission samples above one indoor and four outdoor artificial turf fields posed no significant increase of health risk compared to a single natural grass field, and (ii) the 27 discovered chemicals of concern were found within many of the recommended air risk standards for human exposure (Ginsberg et al., 2011). A health risk evaluation from Canada in 2015 used a selection of governmental reports from the USA, Europe, and Australia to assess the current situation. It reported nine examples of assessments that determined the fields pose low risk to users and one example from Sweden that indicated a low risk level but nonetheless still recommended a ban on the utilization of recycled tire crumb rubber as artificial turf field infill (Toronto Public Health, 2015). Conversely, a preliminary study out of Italy in 2014 concluded that the air vapor produced by heated artificial turf components (common on high temperature days) was of notable concern through the pathways of inhalation, absorption, and digestion (Marsili et al., 2014). The authors determined that a wide variety of harmful chemicals are emitted when the infill components are heated (including polycyclic aromatic hydrocarbons (PAHs) and heavy metals), making public use of these fields unsafe (Marsili et al., 2014). These conflicting human health risk studies emphasize the need for more comprehensive investigations. Such studies concerning chemicals in artificial turf fields are critical because they will form a foundation upon which policy-makers can make informed decisions when developing regulations.

This paper aims to review the international policies surrounding artificial turf and its crumb rubber infill. Regulations from the regions of the EU, United Kingdom (UK), USA, Canada, China, Qatar, and the Global Stockholm Convention are discussed.

## Methods

The countries and regions included in this study were selected to provide a partial representation of the current regulatory status in Asia, Europe, the Middle East, North America, and any globally-impacting agreements.

Information was collected from published papers, technical and policy reports, and the grey literature. These were then analyzed by a collaborative group familiar with the environmental policies in their respective countries in order to extract the pertinent information (e.g., specific chemical limitations and artificial turf field restrictions) from often long, detailed reports. These individuals were primarily identified through their involvement in publications pertinent to artificial turf and crumb rubber infill health research, indicating a familiarity with the chemicals of concern and capability of understanding the existing regulations around these chemicals. They included environmental health professors, active researchers, and governmental agency officials. Much information on policies was taken directly from reports provided to the public by various governmental agencies responsible for their countries’ regulations, such as the ECHA for the EU and the US EPA for the USA; these were commonly available on the respective agency’s website or online archives. The search for pertinent policies was undertaken between May 2021 and January 2022. The analyzed documents and reports were primarily published in English, with some having been translated from their original language. Some documents, such as policies from China, were received in their original language and analyzed by the members of the collaborative group able to report the findings in English. Limitations of this study include the difficulty in locating and accessing governmental policies and regulations, particularly if the primary language of the country was not English. This meant that the policy collection may not be completely exhaustive in its entirety, and some pertinent policies may exist that are not considered herein.

## Results

### European Union (EU)

To understand the progression of the EU’s artificial turf regulations, a brief summary of organizational hierarchy is required. The Registration, Evaluation, Authorization, and Restriction of Chemicals (REACH) was an EU law created in 2006 that requires the registration of chemicals synthesized in or imported into the EU and established the European Chemicals Agency (ECHA) (European Chemicals Agency, 2021). The governing bodies in the EU can use REACH to restrict the permitted amounts of certain hazardous chemicals or to ban them outright, if necessary. REACH sets ‘minimum’ standards that member states and manufacturers may choose to further restrict, if they are so inclined. The ECHA applies approved chemical legislations and contains the Committee for Risk Assessment (RAC) and the Committee for Socio-Economic Analysis (SEAC). The RAC and SEAC advise the European Commission on potential policies concerning the posed substance risk to human and environmental health and the socio-economic implications of such policies, respectively. When chemical regulatory legislation is proposed by the European Commission, ratified by the EU member states represented in the REACH Committee, and approved by the European Parliament and the Council of the European Union, it is then in effect for all 27 member countries of the EU.

The EU’s efforts have been the most ambitious in mitigating the potential human and environmental health effects posed by artificial turf. The two primary target areas for such efforts have focused on the PAH and microplastic content. With regard to PAHs, in March 2018, the ECHA published a record specifying the EU regulation of eight PAHs of concern: benzo[a]pyrene (BaP), dibenzo[a,h]anthracene (DBahA), benzo[a]anthracene (BaA), chrysene (CHR), benzo[j]fluoranthene (BjFA), benzo[b]fluoranthene (BbFA), benzo[k]fluoranthene (BkFA), and benzo[e]pyrene (BeP) (European Chemicals Agency, 2018). The limit on each individual PAH was set to 1 mg/kg (or 1 ppm) for products with plastic or rubber components that have repeated or extended contact with consumer skin or oral cavities (European Chemicals Agency, 2018). The document specified that this regulation only applied to the upper layer of exposed turf fibers in artificial turf (i.e., the “grass” portion); it notably excluded the infill as it is considered a mixture and regulated as such (European Chemicals Agency, 2018). Interestingly, a recent study analyzing crumb rubber infill samples from 91 artificial turf fields in 17 countries found most samples to exceed the 1 mg/kg standard in total amount of the eight PAHs specified in the ECHA report (Armada et al., 2022). The presiding limitation at the time of the EU’s eight PAH specification had been 100 mg/kg for BaP and DBahA and 1000 mg/kg for the other six PAHs (Commission regulation (EU), 2021). Finding the total concentration is more complex than simply summing the individual levels, and the total permitted concentration was thus calculated to be roughly 387 mg/kg for the eight PAHs (depending on the relative contributions of the individual chemicals) (Commission regulation (EU), 2021). The Dutch National Institute for Public Health and Safety (RIVM) and the ECHA had determined in 2017 that this total concentration was too high to definitively assure the safety of members of the public using artificial turf fields with crumb rubber infill and subsequently recommended a lowered standard (Commission regulation (EU), 2021). In June 2019, driven by the initiative of the RIVM, the RAC issued a PAH restriction opinion suggesting a limit of the combined (or total) concentration of the eight PAHs to 20 mg/kg for mixtures supplied to the general public [ ((Commission regulation (EU), 2021, Committee for Risk Assessment, 2019))]. The SEAC published an opinion in September of the same year that supported the Dutch-proposed regulation as modified by the RAC, while emphasizing support for the recommended 12-month transition period before implementation [ ((Commission regulation (EU), 2021, Committee for Risk Assessment, 2019))]. In July 2021, the European Commission followed the opinions of the RAC and the SEAC and passed the 20 mg/kg restriction on the total concentration of eight PAHs used in artificial turf infill and the rubber components of playgrounds; this law will be in effect from August 2022 (Commission regulation (EU), 2021). In January 2010, the EU instituted a 1 mg/kg BaP limit and a 10 mg/kg total PAH limit in the extender oils used for tire production (relevant because end-of-life tires are used to generate turf infill, although not the same as a limit of those concentrations in the actual infill itself) (European Chemicals Agency, 2010). This restriction applies to extender oils used for the production of tires within the EU. However, it is worth noting that it is impossible to guarantee that all crumb rubber infill used in the EU is generated from tires that abide by this regulation. It is important to appreciate that heavy metal elements such as zinc, lead, cadmium, and manganese are also present in tires (and the crumb rubber derived therefrom) (Gomes et al., 2021), and yet no policies currently exist to limit the concentrations of these chemicals in artificial turf infill.

No governing regulation has yet been passed with regard to microplastics. Nevertheless, in January 2019, the ECHA proposed a sweeping restriction on the use of microplastics in EU market products of all types (European Chemicals Agency, 2021). In June 2020, the RAC issued an opinion supporting this restriction and recommended a ban on all microplastics utilized in infill for artificial turf fields after a transition period of six years (Committee for Risk Assessment, 2020). As another option, the RAC also proposed limiting the annual release of microplastics in the fields to 7g/m^2^ (50kg/full sized field), although it stated a clear preference for the complete ban on the use of microplastics in turf infill (Committee for Risk Assessment, 2020). The SEAC followed suit in December 2020 with a supporting opinion that differed in minor recommendations, the most notable of which was the establishment of a 1nm lower size limit for microplastic restrictions (the RAC advised that no lower limit size be established) (Committee for Risk Assessment, 2020). At the time of writing, this proposed microplastics ban has not yet made its way through the extensive EU legislative process. Whether it will be adopted is subject to speculation. However, it is worth noting that the EU has seemed to follow the opinion of its scientific committees in the past with regard to artificial turf restrictions. In March 2020, the European Committee of Standardization (a non-profit organization with no association to the ECHA) generated a report comprising general information for keeping artificial turf fibers and infill within their originally designated areas (European Committee for Standardization, 2020). While not law, the document provides designs, parameters, and general recommendations for minimizing infill microplastic spread to the environ-ent. Individual EU countries have developed similar guidebooks, with the Italian Standardization Body (UNI) (in December 2020) publishing a set of instructions for containing the microplastic content of artificial turf fields within the desired zones (Italian Standardization Body, 2020). Despite the lack of official enforcement of such guidelines, their publication reflects the increased attention being paid to the potential environmental threats posed by artificial turf infill pollution.

The EU has also worked to regulate harmful perfluoroalkyl and polyfluoroalkyl substances (PFAS). The EU’s existing Persistent Organic Pollutants (POPs) regulation was expanded in 2009 to restrict the use of perfluorooctane sulfonic acid (PFOS) to 1 *μ*g/m^2^ in textiles and coated materials and to 10 mg/kg in substances (European Chemcals Agency, 2021). As of July 2020, perfluorooctanoic acid (PFOA) has been banned by the same EU POP regulation (European Chemicals Agency, 2021). After the restriction on PFOA was passed into EU law, concern was soon raised over the use of another PFAS subclass, perfluorohexane-1-sulphonic acid (PFHxS), as a subsequent alternative. Both the SEAC and the RAC issued opinions in 2020 supporting limitation of PFHxS use in the EU, meaning that the onus is on the European Commission to draft an official policy proposal for review (European Chemicals Agency, 2021; European Chemicals Agency, 2021). In January 2020, perfluorobutane-1-sulphonic acid (PFBS) was placed on the REACH chemical list of substances of very high concern (European Chemicals Agency, 2021). This means that the chemical is considered worthy of equal caution as shown to carcinogens, reprotoxicants, and mutagens. Concern has also been raised over the problem of substituting a banned PFAS chemical with another harmful PFAS chemical in the market, just as with the case of replacing PFOA with PFHxS. Due to this issue and the enormous number of substances in the PFAS family, the Netherlands is heading an EU proposal with Belgium, Denmark, Germany, Norway, and Sweden to implement a complete ban on the entire group of PFAS chemicals (Dutch National Institute for Public Health and the Environment, 2021). The proposal, once created, will be submitted to the ECHA to allow the RAC and the SEAC to publish opinions, then be presented to the European Commission for possible adoption to an official policy proposal. While regulatory attenion has been paid to PFAS chemicals, it would appear that policymakers have not considered the health implications of their presence in artificial turf fields.

### United Kingdom (UK)

The withdrawal of the UK from the EU (Brexit) in December 2020 caused many operational and policy complications, including how the UK environmental regulation system would operate. Instead of complying with EU REACH chemical regulations, the new UK REACH now acts to set and enforce such limits (Health and Safety Executive, 2021). While the EU and UK REACH operate independently of each other, the UK REACH was, in principle, founded to maintain the key principles of its EU counterpart. Although the UK is free to maintain current EU standards and follow future EU regulations, the EU-UK Trade and Cooperation Agreement (TCA) (established in December 2020) allows the UK to establish its own regulations (UK-EU Trade and Cooperation Agreement, 2020). The TCA generally states a mutual EU-UK desire for increased environmental protection (UK-EU Trade and Cooperation Agreement, 2020). Whether the UK level of commitment matches that of the EU on artificial turf regulation will be put to the test in the UK response to the new and proposed EU infill regulations of late 2020 and mid-2021. The implementation of Brexit prior to the passing of the EU 20 mg/kg total PAH concentration limit in July 2021 means that the UK is not necessarily bound by these same infill limitations, including any future regulations that may stem from the 2020 ECHA/RAC/SEAC opinion on either an artificial turf infill ban (due to contained microplas-tics) or a limit on annual release of such infill. The Level Playing Field (LPF) provisions of the TCA established areas of non-regression, stating that the “levels of protection provided overall” in these areas cannot be lowered in a way that impacts trade and investment between the EU and UK, if the UK chooses to honor such agreements (UK-EU Trade And Cooperation Agreement, 2020). Potentially pertinent LPF areas for artificial turf policy include climate protection, waste management, and the prevention, reduction, and limitation of risks to human health or the environment arising from the production, use, release, or disposal of chemical substances (UK-EU Trade and Cooperation Agreement. 2020). The rebalancing provisions of the TCA aim to keep the EU and UK at the same general pace for environmental regulations by allowing one side to impose tariffs or other “rebalancing” actions to even the field if one side significantly diverges from (or lags behind) the other with regard to such regulations (UK-EU Trade and Cooperation Agreement, 2020). The strength and effectiveness of these LPF and rebalancing provisions could also be considered questionable given the vague language and limiting influence of “impacting trade and investment” (UK-EU Trade and Cooperation Agreement, 2020).

At the time of writing, the UK REACH limitations relating to artificial turf PAH regulations only include the same EU limits on extender oils used in tire manufacturing and the same 1 mg/kg PAH limit on products supplied to the general public that come into direct or prolonged contact with the human skin or oral cavity (Health and Safety Executive, 2021). There is no active legislation on the limitation of microplastics in artificial turf, even though the Environment Agency (an English environmental protection body) published a quality protocol in 2009 stating that no routes should exist for crumb rubber water runoff from the artificial turf fields (Environment Agency, 2009). The protocol also listed concern for infill that migrates from the field to the outside environment and noted that it should be prevented when practical (Environment Agency, 2009). Due to a lack of concrete parameters and enforcement for the listed concerns, the protocol did very little to advance artificial turf regulation other than documenting early concern for the threat posed by infill to the outside environment.

In 2018, the UK Government approved a ban on all microplastics used in the composition of cosmetic and personal care products (Department for Environment Food and Rural Affairs, 2021). While the rationale for the law stated a strong commitment to prevent these microplastics from entering the outside environment, the UK Government has made little effort to prevent microplastic pollution caused by the crumb rubber infill.

The UK appears to have the same regulatory stance on PFAS chemicals as that of the EU before Brexit in 2020.

Adding to the already-complicated UK regulatory network, individual countries within the UK are free to establish their own environmental regulations, provided that they at least meet the minimum UK REACH requirements. To provide an example of this, one could consider Scotland’s stance relative to that of the UK. The government of Scotland has previously indicated that it plans on upholding EU environmental standards after leaving the EU, and aims to use the UK Withdrawal from the European Union (Continuity) (Scotland) Act 2021 to follow EU regulations when they see fit [ ((UK withdrawal from the European Union (continuity) (Scotland) act, 2021 2021, The Scottish Parliament, 2021))]. This establishes a freedom to abstain from adopting potentially controversial and ambitious environmental regulations, such as the ECHA/RAC/SEAC proposed ban on artificial turf infill (as it does for the other UK countries). The Scottish and UK regulations relating to artificial turf infill chemicals show great variation from regulatory body to regulatory body, without any presiding incentive from the central governments or, generally, the local governments to utilize either sustainable artificial fields or natural grass fields (Watterson, 2017). The Scottish Government has not shown any interest in regulating the crumb rubber infill chemical situation beyond their acknowledgement that artificial turf infill is a microplastic with harmful effects when spread to the environment outside playing fields (Scottish Government, 2021). Notably, this recognition of the environmental threat posed by infill primarily stemmed from their ongoing concern about the levels of crumb rubber in marine litter (Scottish Government, 2021).

The Health and Safety Executive (HSE) monitors Great Britain (England, Scotland, and Wales) for worker health and safely in a variety of spaces, including local authority premises, sports grounds, and school playing fields (Watterson, 2017). It also covers the rubber and plastics industry. In the period 2007 to 2017, the HSE had not reported any checks, tests, or enforcement actions on the chemicals (covered by EU regulations) contained in artificial turf infill and fibers (Watterson, 2017). Since 2017, the HSE has not conducted any tests on the crumb rubber dust and fumes to which artificial turf athletes may be exposed (Watterson, 2017). Nevertheless, the HSE has produced guides for the handling of artificial turf surfaces and crumb rubber infill by artificial turf installers. However, the guides do not address the potential for exposure from inhalation or skin contact to artificial turf and crumb rubber infill materials and therefore do not properly convey the health risks posed to workers (Watterson, 2017).

### United States of America (USA)

Environmental regulations in the USA can be implemented at the federal, state, and local levels. The regulatory network of the federal government concerning artificial turf and its crumb rubber infill is virtually nonexistent, as is regulation of the notable chemicals contained therein. The gap between the USA and the EU is best demonstrated in the regulations (or lack thereof) concerning the PAH chemicals from which crumb rubber infill is composed. PAH content is regulated in the US by many agencies including the American Conference of Governmental Industrial Hygienists, the National Institute for Occupational Safety and Health (NIOSH), the Occupational Safety and Health Administration (OSHA), and most notably the US Environmental Protection Agency (US EPA) (Agency for Toxic Substances and Disease Registry, 2021). While several agencies consider PAHs, the only existing regulations at the time of writing concern PAH concentrations in the air and drinking water; there are no clear concentration limits for solid products or mixtures, such as crumb rubber (Agency for Toxic Substances and Disease Registry, 2021; United States Environmental Protection Agency, 1996). The extent of federal government attention to the issue of artificial turf and crumb rubber infill has consisted of funding a multi-agency research initiative, the Federal Research Action Plan on Recycled Tire Crumb Used on Playing Fields and Playgrounds, in February 2016. This resulted in a research report developed by the US Center for Disease Control and Prevention (US CDC), the Agency for Toxic Substances and Disease Registry (ATSDR), the Consumer Product Safety Commission (CPSC), and the US EPA (United States Environmental Protection Agency, 2021). The report was to be released in two parts: the first characterizes chemicals associated with crumb rubber, and the second evaluates the potential human exposures to such chemicals during use of an artificial turf field (United States Environmental Protection Agency, 2021). This plan was created, in part, to help guide US policy makers in their regulation of crumb rubber infill. Part 1 was released in July 2019 (Agency for Toxic Substances and Disease Registry, 2019; Agency for Toxic Substances and Disease Registry, 2019) and Part 2 has not been published at the time of writing.

The USA similarly has no direct regulations in place for concentrations of heavy metals in crumb rubber infill. Concerns about the levels of zinc in the artificial turf infill have been raised in recent years, with one study detecting an upper value of 14,150 ± 1344 mg/kg in the crumb rubber (Gomes et al., 2021; Celeiro et al., 2018). These findings have not yet translated into policy as the levels fall within established limits for soil. For reference, the US EPA has set the urban and rural surface soil concentration limit for zinc at 23,000 mg/kg (Gomes et al., 2021). It should be noted, however, that human exposure to chemicals in soil differs from that in crumb rubber on artificial turf fields because repeated and prolonged skin-to-concentrated infill contact occurs when people engage in activities on artificial turf fields. The acceptable chemical concentration levels for soil and turf should thus be assessed independently of each other and take into account the extent and duration of exposure.

The USA has paid considerable attention to PFAS chemicals that pose a potential threat to human and environmental health. The EPA uses the Toxic Substances Control Act (TSCA) to analyze and control harmful PFAS chemicals (United States Environmental Protection Agency, 2021). A large number of PFAS substances have been reported for use to the EPA by product manufacturers, and many of them have been subjected to some form of regulation. However, for the most part, such regulation consists of testing with no limitations on production or use. In 2021, the EPA began to implement a new roadmap to address PFAS chemicals, marked primarily by plans of an organized national testing protocol, updated analytical profiles for certain PFAS chemicals, and increased national monitoring (United States Environmental Protection Agency, 2021). It is possible that these increased efforts will lead to concrete restrictions in the future. For PFAS chemicals of higher concern to human health and the environment, the testing is required to be more rigorous and the chemicals must be approved before they are permitted to enter the market (United States Environmental Protection Agency, 2021; United States Environmental Protection Agency, 2021).

In 2020, the Massachusetts Toxics Use Reduction Institute (TURI) published a PFAS background report that included two studies showing the presence of numerous PFAS chemicals of concern (including PFOS, PFOA, and PFBS) in artificial turf fibers and backing (Massachusetts Toxics Use Reduction Institute, 2020). Both studies appear to have been preliminary and did not report statistics beyond detection of presence, such as the range or maximum concentrations. In 2006, the EPA initiated a program with eight leading PFAS manufacturing companies to phase out the intentional synthesis of PFOA in products and emissions by 2015 (United States Environmental Protection Agency, 2021). All participating companies met the goal. However, PFOA may still be unintentionally emitted into products in its short-chain form (United States Environmental Protection Agency, 2022). As of January 2015, any company knowingly manufacturing or processing PFOA was required to notify the EPA and allow 90 days for preliminary risk assessment and subsequent regulation if deemed necessary (United States Environmental Protection Agency, 2021). In July 2020, the EPA issued a directive that any company or manufacturer must notify and receive approval from the EPA before any recontinuation of use of long-chain PFAS chemicals, such as PFOA and PFOS (United States Environmental Protection Agency, 2021). The directive also requires EPA review before products with a surface coating of a set group of long-chain PFAS or carpets containing perfluoroalkyl sulfonate can be imported to the US (United States Environmental Protection Agency, 2021).

In December 2021, the EPA issued a public health advisory for both PFOA and PFOS (United States Environmental Protection Agency, 2021). However, the tightened regulation of intentional PFOS and PFOA manufacture (as previously discussed) does not mean that the potential for human exposure to these PFAS chemicals no longer exists. For example, any artificial turf surface that was either installed before the increased regulations or unintentionally contaminated by PFAS chemicals in production may still contain these chemicals or any number of unrestricted PFAS family chemicals. This raises the potential concern over the public use of neglected fields that have not been replaced since the EPA addressal of the PFOS threat. These PFAS regulations largely avoid banning any subset of PFAS chemicals, but rather bring their manufacture and use under the discretion of the EPA. It is unclear whether these PFAS regulations apply to artificial turf fibers and crumb rubber infill or not. If they do apply, it is unclear whether they have been enforced through testing of the artificial turf and crumb rubber, as there appears to be no ruling policies or regulations regarding mandatory testing of these products before or after public installation. Individual US states may have more restrictive controls. For example, in July 2021, the California EPA listed carpets and rugs containing PFAS chemicals to be priority substances, meaning that they are recognized to have a hazard trait that can harm people or the environment (California Environmental Protection Agency, 2020). However, artificial turf was explicitly and most notably excluded from this piece of legislation (California Environmental Protection Agency, 2020).

In 2015, the US Government passed the Microbead-Free Waters Act, which banned the use of cosmetic products containing microplastics (United States Food and Drug Administration, 2021). In this law (which applies only to cosmetics), a microplastic was defined as any solid plastic unit smaller than 5mm in size (United States Food and Drug Administration, 2021). The law also uses the same rationale as that of the 2018 UK ban on cosmetic microplastics, i.e., serious concern over the environmental damage caused by these products. Paradoxically, the US Government has not regulated microplastics in crumb rubber infill, even though the pollutant potential from this source is great.

In terms of direct policy, the majority of the USA (at the federal, state, or local level) has no existing regulations concerning artificial turf and crumb rubber. Nevertheless, the issue has been gaining attention, and dispersed throughout the country are areas that have implemented policies favoring fields with sustainable alternatives to crumb rubber infill. Leading the way in 2009, the Los Angeles Unified School District placed a ban on synthetic turf fields containing crumb rubber infill, and required all newly installed turf fields to utilize alternative infill materials (California State Legislature, 2016). In 2015, Edmonds City Council in Washington passed a 30 month moratorium on the installation of artificial turf fields containing crumb rubber infill (Edmonds City Council, 2015). The Hartford City Council in Connecticut implemented a zoning regulation that bans the use of crumb rubber in all fields installed after January 2016 (Hartford City Council, 2018). In 2017, Washington, DC introduced a moratorium on the installation of any synthetic turf field utilizing crumb rubber infill (Council of the District of Columbia, 2017). Adjacent to Washington, DC, Montgomery County in Maryland (the state’s most populated region) also passed a ban on crumb rubber field installations (Monahan, Rappleye, and Gosk, 2021). In 2018, Westport, Connecticut banned the installation of turf fields containing crumb rubber and, in the following year, allocated $4.7 million for the replacement of four such fields in favor of those with sustainable infill alternatives not made from recycles tires (Vaughan, 2021; Vaughan, 2021). Lastly, the New York City Department of Parks and Recreation has released a directive stating that all newly installed fields must utilize infill methods other than crumb rubber (NYC Health, 2021). It is important to note that all listed US policies only apply to turf fields installed after the date of release, meaning that all crumb rubber infill fields installed before the implementation of these policies were permitted to remain and be utilized by the public.

In summary, although the USA recognizes microplastics and the PAH and PFAS chemicals contained in artificial turf fibers and crumb rubber infill to be harmful to human and environmental health, no federal policies have been developed and implemented that directly regulate the installation or chemical composition of artificial turf fields. Similarly, the vast majority of state and local governments have established no regulations. The lack of legislative action likely stems from the absence of conclusive studies demonstrating that average use of the artificial turf fields leads to adverse human health effects. With the conduct of more comprehensive and focused investigations, it is anticipated that part 2 of the Federal Research Action Plan on Recycled Tire Crumb Used on Playing Fields and Playgrounds will shed more light on the health risks of artificial turf fields and provide a foundation for the development of additional policies.

### Canada

The Canadian government has not established any standards that directly regulate artificial turf fields or crumb rubber infill. The Canadian Environmental Protection Act (CEPA) (1999) defined a toxic substance as one that enters or may enter the environment under concentrations or conditions that may have an immediate or long-term harmful effect on the environment, may constitute a danger to the environment on which life depends, or may constitute a danger in Canada to human life or health (Government of Canada, 2005). When a substance is labeled as toxic in Canada, at least one method of preventing its entrance to the environment must be implemented. Common methods include regulations, pollution prevention plans, environmental emergency plans, environmental codes of practice, and environmental release guidelines (Government of Canada, 2005). Distillate aromatic extracts (DAEs) are aromatic extract substances created as a side product in the refinement of crude oil and are known to contain notable concentrations of PAHs (Government of, 2021). In August 2017, the government of Canada released an assessment of three potentially harmful DAEs found in crumb rubber infill, and determined that the three DAEs were not a risk to environmental or human health (Health Canada August, 2017). On the basis of this report, the government determined crumb rubber to not be classified as a toxic substance (Health Canada August, 2017). They also recommended that no further action be taken to regulate crumb rubber on account of these DAEs (Health Canada August, 2017). It is also noted that in the EU, regulations restrict the use of DAEs in tires. In Canada, tire manufacturers no longer use DAEs (Health Canada August, 2017).

Although the PAH chemical regulations in Canada appear to differ between jurisdictions, there are no established limits on solid products or mixtures (such as artificial turf fibers and crumb rubber infill) (CAREX Canada, 2021). A total PAH occupational exposure limit of 0.2 mg/m^3^ has been placed, along with limits on air emissions, drinking water and food contamination, and (in British Columbia only) on soil standards (CAREX Canada, 2021). Because no established limits have been set for PAHs in solid products, the current PAH chemical regulations appear to not apply to artificial turf fibers or crumb rubber.

In June 2017, Canada released the Microbeads in Toiletries Regulations, placing a near-identical ban on the sale, manufacture, and import of cosmetic microplastics as that of the USA and the UK (Government of Canada, 2021). As was the case for the other two regions, no limits have been placed on microplastics released into the environment from artificial turf fields.

Canada does not manufacture PFAS chemicals. However, they may be imported into the country (O’Keeffe, 2021). Canada placed a ban on the import and use of PFOS in 2008, with exceptions being made for use in firefighting foam, the military, and some photographic media (O’Keeffe, 2021). In addition, PFOA and long-chain perfluorocarboxylic acids (PFCAs) have been banned through regulations, with a similar concern being raised over the potential replacements for these common PFAS as seen in other regulatory jurisdictions (such as the EU) (Government of Canada, 2021). Similar to the USA, Canada in 2021 announced a renewed focus on PFAS chemicals that primarily features continued research, potential policies addressing PFAS as a chemical family (thus mitigating the hazards of the numerous potential substitutes for banned substances), and a review of policies from other governmental bodies to provide guidance on potential courses of action (Government of Canada, 2021). Like the USA, Canada has not yet made any regulatory connection between PFAS and the risk they pose to the public through exposure to artificial turf fields containing such chemicals.

### China

China issued the national standard GB/T 20394-2019 in 2019, stipulating the release limits of migratory elements and harmful substances in artificial grass products for sports. The migratable elements specified were antimony, arsenic, barium, cadmium, chromium, lead, mercury and selenium with the maximum limits being 60, 25, 1000, 75, 60, 90, 60, and 500 mg/kg, respectively (The Standardization Administration of China, 2019). Hazardous substances include total volatile organic compounds (VOCs), styrene, formaldehyde, and 4-phenylcyclohexane with maximum limits of 0.600, 0.500, 0.050, and 0.050 mg/(m^2^•h), respectively (The Standardization Administration of China, 2019).

Vulcanized rubber powder is a product of waste rubber recycling, and it can be used in the production of recycled rubber, rubber products and laying rubber runway (amongst many other uses) (Fazli and Rodrigue, 2020). In 2020, China passed the national standard GB/T19208-2020, that stipulates the limit of PAHs and hazardous substances that may be contained in vulcanized rubber powder. The limits of 18 kinds of PAHs are divided into three levels, namely 150 mg/kg for level I, 200 mg/kg for level II, and 300 mg/kg for level III (The Standardization Administration of China, 2020). The limit of BaP is 20 mg/kg. The limits of polybrominated biphenyls (PBBs), polybrominated diphenyl ethers (PBDEs), lead, mercury, and hexavalent chromium are all 1000 mg/kg. The limit of cadmium is 100 mg/kg (The Standardization Administration of China, 2020).

At the time of writing, it would appear that no standards have been established by the Chinese Government concerning the concentration of PFAS chemicals in artificial turf and crumb rubber infill. Similarly, no policies appear to have been created to address the issue of microplastics in these products.

### Qatar

There appears to be no regulatory network of the Qatar Government surrounding artificial turf fields and crumb rubber infill. In April 2021, Qatar released the Ashghal Recycling Manual, which was created, in part, to detail the specifications and specific criteria for generation of crumb rubber from recycled tires. This document outlined thorough and extensive standards for the structure, coating, performance, and quality of the crumb rubber granules, but it did not provide limitations for any chemicals of concern (such as PAHs, PFAS, VOCs, or heavy metals) (Ashghal Public Works Authority, 2021). Our search found no regulations in Qatar concerning permissible levels of PAHs, PFAS, VOCs, or heavy metals in crumb rubber, artificial turf fields, or human exposure. According to Integral, an artificial turf manufacturer, the artificial turf fields and crumb rubber infill are utilized widely in Qatar and are commonplace in sports fields and facilities (Integral Grass. Carpets of artificial grass in Qatar, 2022).

In 1979, Qatar joined the governments of Bahrain, Iran, Iraq, Kuwait, Oman, Saudi Arabia, and the United Arab Emirates in creating the Regional Organization for the Protection of the Marine Environment (ROPME). ROPME was formed, in part, to enforce the Kuwait Regional Convention for Cooperation on the Protection of the Marine Environment from Pollution, which was also signed by Qatar in 1979 (The Regional Organization for the Protection of the Marine Environment, 1978). This Regional Convention stated a general commitment to preventing marine pollutant waste and to the development of guidelines to prevent major pollution from future projects (The Regional Organization for the Protection of the Marine Environment ,1978). ROPME has since developed specific protocols pertinent to the original Convention, such as the 1990 Protocol for the Protection of the Marine Environment against Pollution from Land-Based Sources, which is an agreement by all parties to develop regional environmental standards, conduct routine assessments of marine health, identify the major sources pollution, and to generally prevent heavy pollution to the marine environment (The Regional Organization for the Protection of the Marine Environment, 1990). The extent to which Qatar has executed concrete actions to meet the intentions of these accords is unknown to the authors, although our search for related policies has not located any such regulations. Despite their stated commitment to marine health, granular and fibrous microplastics have been found in the waters of Qatar’s Exclusive Economic Zone (Castillo et al., 2016). These microplastics were detected in an average concentration of 0.71 particles/m^3^ (Castillo et al., 2016). While such microplastic pollution is attributable to a large number of possible sources, the serious potential for a contribution from the crumb rubber infill of artificial turf fields should not be overlooked. At the time of writing Qatar has not created any regulations concerning microplastics in the artificial turf fields or otherwise.

Qatar is recently of particular note regarding athletics safety because it is serving as host of the upcoming 2022 FIFA World Cup.

### Global

The Stockholm Convention is an agreement created by the United Nations (UN) in 2004 and signed by over 152 countries to control the release of persistent organic pollutants (United Nations Industrial Development Organization (UNIDO), 2021). Germane to artificial turf, two PFAS chemical groups (PFOA and PFOS) have been included in the official listings, while an additional compound, PFHxS, is currently under review for restriction. In 2019, PFOA was incorporated into the Stockholm Convention list of chemicals for elimination of use (acting effectively as a ban), while in 2009, PFOS was included on the list of restricted use (meaning that there are increased regulations without a full ban) (European Chemicals Agency, 2021). In 2017, Norway submitted a request to place PFHxS under Stockholm Convention regulations, and the proposal is still being considered at the time of writing (European Chemicals Agency, 2021). Notable countries that have signed but not ratified (and thus not placed into effect) the Stockholm Convention include the USA, Italy, and Israel (United Nations Environment Programme, 2021).

A summary of the main relevant regulations for countries discussed in this review is provided in Table 1, and a list of the chemicals addressed therein is included in Table 2.

The regulations listed are only those pertinent to the topic of artificial turf fields and crumb rubber infill. These regulations are not exhaustive, but are a collection of notable policies relevant to the topic. A label of “none” indicates that our search failed to locate any policies pertinent to the regulatory area. Abbreviations: polycyclic aromatic hydrocarbons (PAHs); perfluoroalkyl and polyfluoroalkyl substances (PFAS); benzo[a]pyrene (BaP); dibenzo[a,h]anthracene (DBahA); benzo[a]anthracene (BaA); chrysene (CHR); benzo[j]fluoranthene (BjFA); benzo[b]fluoranthene (BbFA); benzo[k]fluoranthene (BkFA); benzo[e]pyrene (BeP); perfluorooctane sulfonic acid (PFOS); perfluorooctanoic acid (PFOA); perfluorobutane-1-sulphonic acid (PFBS); The Registration, Evaluation, Authorization, and Restriction of Chemicals (REACH); perfluorohexane-1-sulphonic acid (PFHxS); European Union (EU); Environmental Protection Agency (EPA); perfluorocarboxylic acids (PFCA).

The chemicals listed are only those contained in regulations pertinent to the topic of artificial turf fields and crumb rubber infill. Abbreviations: polycyclic aromatic hydrocarbons (PAH); benzo[a]pyrene (BaP); dibenzo[a,h]anthracene (DBahA); benzo[a]anthracene (BaA); chrysene (CHR); benzo[j]fluoranthene (BjFA); benzo[b]fluoranthene (BbFA); benzo[k]fluoranthene (BkFA); benzo[e]pyrene (BeP); perfluoroalkyl and polyfluoroalkyl substances (PFAS); perfluorooctane sulfonic acid (PFOS); perfluorooctanoic acid (PFOA); perfluorobutane-1-sulphonic acid (PFBS); long-chain perfluorocarboxylic acids (PFCA); perfluorohexane-1-sulphonic acid (PFHxS); antimony (Sb); arsenic (As); barium (Ba); cadmium (Cd); chromium (Cr); lead (Pb); mercury (Hg); selenium (Se); zinc (Zn); volatile organic compounds (VOC); polybrominated biphenyls (PBB); polybrominated diphenyl ethers (PBDE).

## Discussion

Artificial turf and crumb rubber infill contain and elaborate a wide range of substances (e.g., PAHs, microplastics, PFAS, and heavy metals) that can pose a threat to human health and the environment. Given their potential for adverse impacts, one might expect that countries would have established policies that regulate their use and/or limit the extent to which humans or the environment are exposed to them. After a review of the governmental policies relating to artificial turf, crumb rubber infill, and the substances associated with them, it is clear that there is great variability between countries regarding (i) whether the various chemicals are subject to regulation (and if they are, what constitutes acceptable levels), and (ii) whether artificial turf and crumb rubber infill are directly considered for regulation. For example, the EU has the most comprehensive regulations regarding PAHs, microplastics, and PFAS, and includes direct consideration of artificial turf and crumb rubber infill for PAHs and microplastics. Others, like the USA and Canada, have regulations on the substances but have not established exposure limits for artificial turf and crumb rubber infill.

Of the substances subject to governmental regulation, the PAHs have been subjected to the most scrutiny, likely because many of these chemicals are known carcinogens and may elicit serious adverse health effects through exposure (European Chemicals Agency, 2021). Given that artificial turf infill is most commonly created from shredding used tires that contain PAHs, one might expect that the regulations surrounding these chemicals would apply to the infill. This was the case for China, the EU, and the UK. Canada and the USA enacted regulations that established PAH levels for other exposure sources (e.g., air or water), but not for artificial turf or crumb rubber infill.

Compounds closely related to PAHs have also been detected in crumb rubber in the Netherlands, although most have not been regulated in any country (Skoczyńska et al., 1 August 2021). These include substituted and heterocyclic PAHs, e.g., benzothiazoles, benzothiofuranes, benzonaphthothiophenes, and aromatic amines. Concern over the presence of N-(1,3-dimethylbutyl)-N′-phenyl-p-phenylenediamine (6PPD) quinone, N-N’-diphenylguanidine (DPG), and other similar tire rubber oxidation products in end-of-life tire products, such as crumb rubber infill, have also been raised due to their potential for harm to the environment and health (Tian et al., 2021; Challis et al., 2021). 6PPD quinone has been the subject of particular concern due to its toxicity to coho salmon populations through tire rubber runoff (Tian et al., 2021). Despite the large number and wide variety of these chemicals within crumb rubber, very few policies have been created to limit their presence or concentrations.

Microplastics have been identified as products of concern because they are not biodegradable and can thus create bioaccumulation problems in the outside environment (Accessed 7 July 2021). This affects general ecological health and contamination levels of food and drinking water (Accessed 7 July 2021). The ECHA estimates that around 42,000 tons of microplastics pollute the environment annually; escaped artificial turf crumb rubber infill is the largest individual contributor, being responsible for as much as 16,000 tons (Accessed 7 July 2021). For those countries regulating microplastics, the majority (e.g., Canada, the UK, and the USA) only regulate levels in cosmetics and personal products, and do not address crumb rubber infill.

Cadmium, lead, and zinc are present in crumb rubber infill. While such metals can exert negative effects on the environment and human health (Gomes et al., 2021), most Western countries do not regulate their levels in artificial turf or crumb rubber infill. This is concerning, particularly given the well-established adverse influence of lead exposure on the development of children (Delgado et al., 2018; Matte, 2003) and the high frequency of children engaging in activities on artificial turf.

The PFAS family of compounds is of notable concern. Often referred to as “forever chemicals,” PFAS are known for their resistance to degradation in both the environment and biological systems (including the human body), and, relatedly, their persistence and accumulation over exceptionally long periods of time (European Chemicals Agency, 2021; Agency USEP. PFAS explained, 2021). The US CDC reported that PFAS can be detected in the bloodstream of 98% of Americans (Sunderland et al., 2019). This is potentially problematic because members of the PFAS family exhibit severe toxic properties. Many possess immunotoxic effects, elicit damage to reproductive and fetal health, or disrupt the endocrine system, while some exhibit carcinogenic characteristics (European Chemicals Agency, 2021; Agency USEP. PFAS explained, 2021; Schrenk and Bignami, 2020). PFOA, PFOS, and PFBS are PFAS chemicals particularly noted for their adverse health effects (European Chemicals Agency, 2021; Agency USEP. PFAS explained, 2021). These have been associated with artificial turf fibers and backing (Massachusetts Toxics Use Reduction Institute, 2020). Western governments have enacted legislation to regulate these specific chemicals, although limits in artificial turf or crumb rubber infill have not been applied. It is important to appreciate that many other PFAS family members that can elicit adverse health effects (e.g., PFHxS and other closely related subclasses) remain unregulated.

Artificial turf fields (the majority of which use crumb rubber infill) are becoming increasingly prevalent as a low maintenance, consistent surface alternative to grass fields. The routine use of these fields by sportspeople and children makes these populations vulnerable to exposure to the chemicals contained in (or emitted from) the artificial turf and associated infill. This consideration would certainly apply to occupational exposure, such as with workers involved in artificial turf installation and maintenance. Globally, few to no direct policies currently exist that would serve to protect any of these people from artificial turf chemicals. This may relate, at least in part, to insufficient or unreliable information about the health effects of artificial turf (and associated crumb rubber infill) substances. Legislative decision-making in this area is further hindered by a lack of knowledge about adverse human health effects of other crumb rubber substances (e.g., substituted and heterocyclic PAHs such as benzothiazoles, benzothiofuranes, benzonaphthothiophenes, chlorinated paraffins, and aromatic amines) (Skoczyńska et al., 1 August 2021; Brandsma et al., July 2 2019). Clearly, funding is needed to support research investigating (i) the toxicity and health effects of the broad array of artificial turf substances, and (ii) the extent of exposure of artificial turf users and workers to the artificial turf substances. The Stockholm Convention serves as a good example of decision-making based on science. Unfortunately, these processes take time. In the interim, users of artificial turf fields remain unaware about (and largely unprotected by regulatory bodies from) the potential adverse health risks associated with use of the fields. It is hoped that the presentation of policies from different countries provides insights that serve as a catalyst for the deliberate investigation of chemicals in artificial turf and crumb rubber infill, the utilization of “safer” infill materials, and the development of well-founded policies that ensure the continuing health and well-being of users of artificial turf fields.

## Figures and Tables

**Table 1:** The current state of regulations in each region.

Region	Direct Turf/Infill Regulation	PAH Regulation	Microplastics Regulation	PFAS Regulation
European Union	Proposed ban on microplastics used as artificial turf infill (includes crumb rubber infill)	Individual concentration limit of 1mg/kg on eight PAHs of concern in products with repeated or prolonged exposure (BaP, DBAhA, BaA, CHR, BjFA, BbFA, BkFA, BeP) Concentration limit of 20mg/kg for the total of all eight PAHs of concern in mixtures (includes crumb rubber infill)	Proposed ban on microplastics used as artificial turf infill	Ban on PFOS and PFOA; PFBS placed on REACH chemical list of substances of very high concern; Proposed restriction of PFHxS as alternative for PFOA; Draft of proposal for ban of all PFAS substances currently in progress from the Netherlands, Belgium, Germany, Denmark, Norway, and Sweden
United Kingdom	None	Individual concentration limit of 1mg/kg on eight PAHs of concern in products with repeated or prolonged exposure (BaP, DBahA, BaA, CHR, BjFA, BbFA, BkFA, BeP)	Ban on all microplastics used in the composition of cosmetics and personal care products	Prior to Brexit, the same policies as the EU; Post-Brexit policies unclear at time of writing
United States of America	No regulations from the central government; Individual regions of Los Angeles, Edmonds, Hartford, Washington DC, Montgomery, Westport, and New York City have implemented restrictions primarily concerning artificial turf fields utilizing crumb rubber infill	No concentration limits for solid products or mixtures	Ban on all microplastics used in the composition of cosmetics and personal care products	PFOA and PFOS restricted through EPA review in manufacture and import
Canada	None	No concentration limits for solid products or mixtures; General total PAH occupational exposure limit of 0.2 mg/m^3^	Ban on all microplastics used in the composition of cosmetics and personal care products	Does not manufacture PFAS family chemicals; Ban on PFOA and long-chain PFCA
China	None	Limits of 18 types of PAH chemicals based on three categories: 150 mg/kg for Level I PAHs 200 mg/kg for Level II PAHs, 300 mg/kg for Level III PAHs Individual limit of 20 mg/kg for BaP	None	None
Qatar	None	None	None	None

**Table 2:** List of substances addressed through regulatory action.

Substance Class	Specific Substance
PAH	BaP
	DBahA
	BaA
	CHR
	BjFA
	BbFA
	BkFA
	BeP
PFAS	PFOS
	PFOA
	PFBS
	PFCA
	PFHxS
Heavy	Sb
Metals	As
	Ba
	Cd
	Cr
	Pb
	Hg
	Se
	Zn
VOC	Styrene
	Formaldehyde
	4-phenylcyclohexane
Polybrominated	PBB
Substances	PBDE
Microplastics	Not Applicable
